# Prognostic value of fatty acid metabolism-related genes in patients with hepatocellular carcinoma

**DOI:** 10.18632/aging.203288

**Published:** 2021-07-13

**Authors:** Dongsheng He, Lifang Cai, Weiming Huang, Qingqing Weng, Xi Lin, Mengxing You, Shengyin Liao

**Affiliations:** 1 Department of Medical Oncology, The First Hospital of Putian, Teaching Hospital, Fujian Medical University, Putian 351100, Fujian Province, China

**Keywords:** hepatocellular carcinoma, TCGA, ICGC, fatty acid metabolism, prognosis

## Abstract

The deregulation of fatty acid metabolism plays a crucial role in cancer. However, the prognostic value of genes involved in the metabolism in hepatocellular carcinoma (HCC) remains largely unknown. We first constructed a multi-fatty acid metabolic gene prognostic model of HCC based on The Cancer Genome Atlas (TCGA) and further validated it using the International Cancer Genome Consortium (ICGC) database. The model was integrated with the clinical parameters, and a nomogram was built and weighted. Moreover, immune cell infiltration of the tumor microenvironment was investigated. A prognostic model was constructed using 6 selected fatty acid metabolism-related genes, and HCC patients were divided into high- and low-risk groups. Receiver operating characteristic curve (ROC) analysis, principal component analysis (PCA), and t-distributed stochastic neighbor embedding (t-SNE) analysis showed the optimal performance of the model. The concordance index (C-index), ROC curve, calibration plot and decision curve analysis (DCA) all confirmed the satisfactory predictive capacity of the nomogram. The analysis of immune cell infiltration in HCC patients revealed a correlation with different risk levels. Our findings indicate that a prognostic model based on fatty acid metabolism-related genes has superior predictive capacities, which provides the possibility for further improving the individualized treatment of patients with HCC.

## INTRODUCTION

Liver cancer is the sixth most common cancer and leads to unacceptable mortality, with more than 800,000 deaths reported in 2020 [1]. As the most common primary component of liver cancer, hepatocellular carcinoma (HCC) has already become a serious global public health problem [2]. Although multiple treatments, such as surgery, chemotherapy, radiotherapy and targeted therapy, have been developed in recent years, the 5-year survival rate of patients with HCC is unsatisfactory due to recurrence in a large proportion of patients after hepatectomy and diagnosis at an advanced stage [3]. Consequently, studies exploring novel therapeutic targets and developing novel prognostic models are urgently needed for patients with HCC.

Metabolic dysregulation is one of the ten hallmarks of cancers, and accumulating evidence indicates that metabolic reprogramming plays a crucial role in the initiation and development of cancer [4–6]. Lipid metabolic reprogramming is one of the most prominent metabolic changes observed in cancer cells and has received increasing attention. As an important component of lipid metabolism, the accumulation of fatty acids has been observed, which can be used to satisfy the requirement for lipids to synthesize signaling molecules and membranes. Fatty acids are required for membrane synthesis, energy storage and the generation of signaling molecules, and accumulating evidence suggests that fatty acids are indispensable for the initiation and development of cancers such as breast cancer and colorectal cancer [7–9]. Moreover, deregulated fatty acids might not only disturb the curative effect of chemotherapeutic and radiation treatments on patients with cancer [10, 11] but also affect immunotherapy, which has been a breakthrough in oncotherapy in recent years. Fatty acids in the tumor microenvironment affect the function and phenotype of infiltrating immune cells, which are associated with immunosuppression [12]. Treatments targeting deregulated fatty acids in cancer might slow tumor growth and exert synergistic effects with immune checkpoint inhibitors [13]. Nevertheless, the distinct fatty acid metabolism-related genes, prognostic value and relationship with immunotherapy in HCC remain largely unknown.

In the present study, we first comprehensively analyzed the correlation between differentially expressed fatty acid metabolism-related genes and the prognosis of patients with HCC and constructed a prognostic model based on The Cancer Genome Atlas (TCGA) database. Then, the prognostic risk model was further validated using the International Cancer Genome Consortium (ICGC) database, and patients with HCC were divided into high- and low-risk groups. Moreover, the prognostic risk model was integrated with the clinical parameters of patients with HCC. Subsequently, a clinical prognostic model of patients with HCC was structured and validated from different perspectives. Finally, the difference in immune cell infiltration in high- and low-risk patients with HCC was explored. Taken together, our findings indicated that fatty acid metabolism-related genes might be potential prognostic markers and therapeutic targets in patients with HCC and further improve the efficacy of treatment in patients with HCC through personalized treatment.

## RESULTS

### Identification of fatty acid metabolism-related genes in patients with HCC

**Figure 1:**
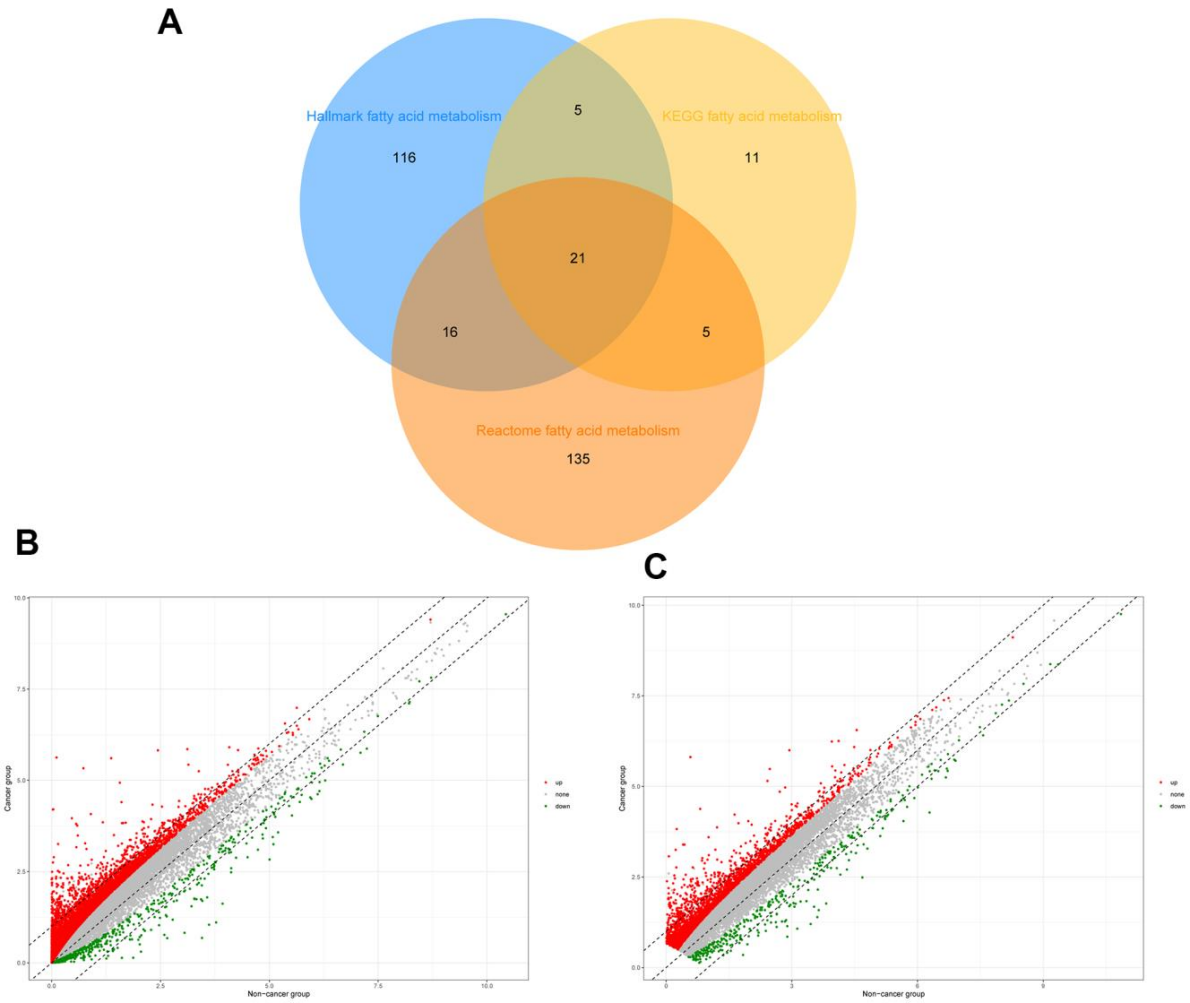
**Identification of differential expressed fatty acid metabolic genes in HCC.** (**A**) Venn diagram of three fatty acid metabolic gene sets. (**B**) Differential expressed genes of HCC patients in TCGA. (**C**) Differential expressed genes of HCC patients in ICGC.

To identify genes related to fatty acid metabolism, the intersection of the three gene sets related to fatty acid metabolism was filtered, and 309 genes were extracted after the overlapping genes were removed (Figure 1A). The “Limma” R package was used to identify 12,450 differentially expressed genes (DEGs) from TCGA and 2,939 DEGs from the ICGC database (Figure 1B, 1C).

**Figure 2:**
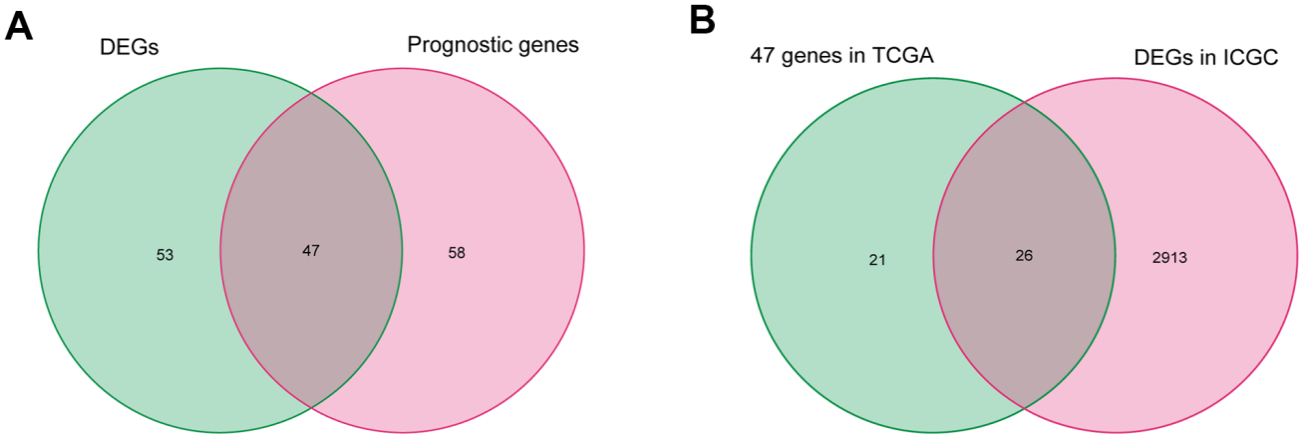
**Identification of differentially expressed fatty acid metabolic genes correlated with OS in HCC.** (**A**) Differentially expressed fatty acid metabolic genes correlated with OS in TCGA. (**B**) Further validation in ICGC.

After excluding data from patients with a follow-up time of less than 90 days, the fatty acid metabolism-related genes and the corresponding expression profile were filtered in 329 patients with HCC in TCGA. First, 105 fatty acid metabolism-related genes correlated with overall survival (OS) were identified by performing a univariate Cox proportional hazard analysis. Next, 47 differentially expressed fatty acid metabolism-related genes correlated with OS were selected by intersecting the DEGs, and 105 fatty acid metabolism-related genes were correlated with the OS of patients in TCGA (Figure 2A). Then, the 47 selected fatty acid metabolism-related genes were validated by analyzing DEGs in the ICGC database, and 26 genes were filtered (Figure 2B).

### Construction and assessment of a prognostic model based on selected fatty acid metabolism-related genes

**Figure 3:**
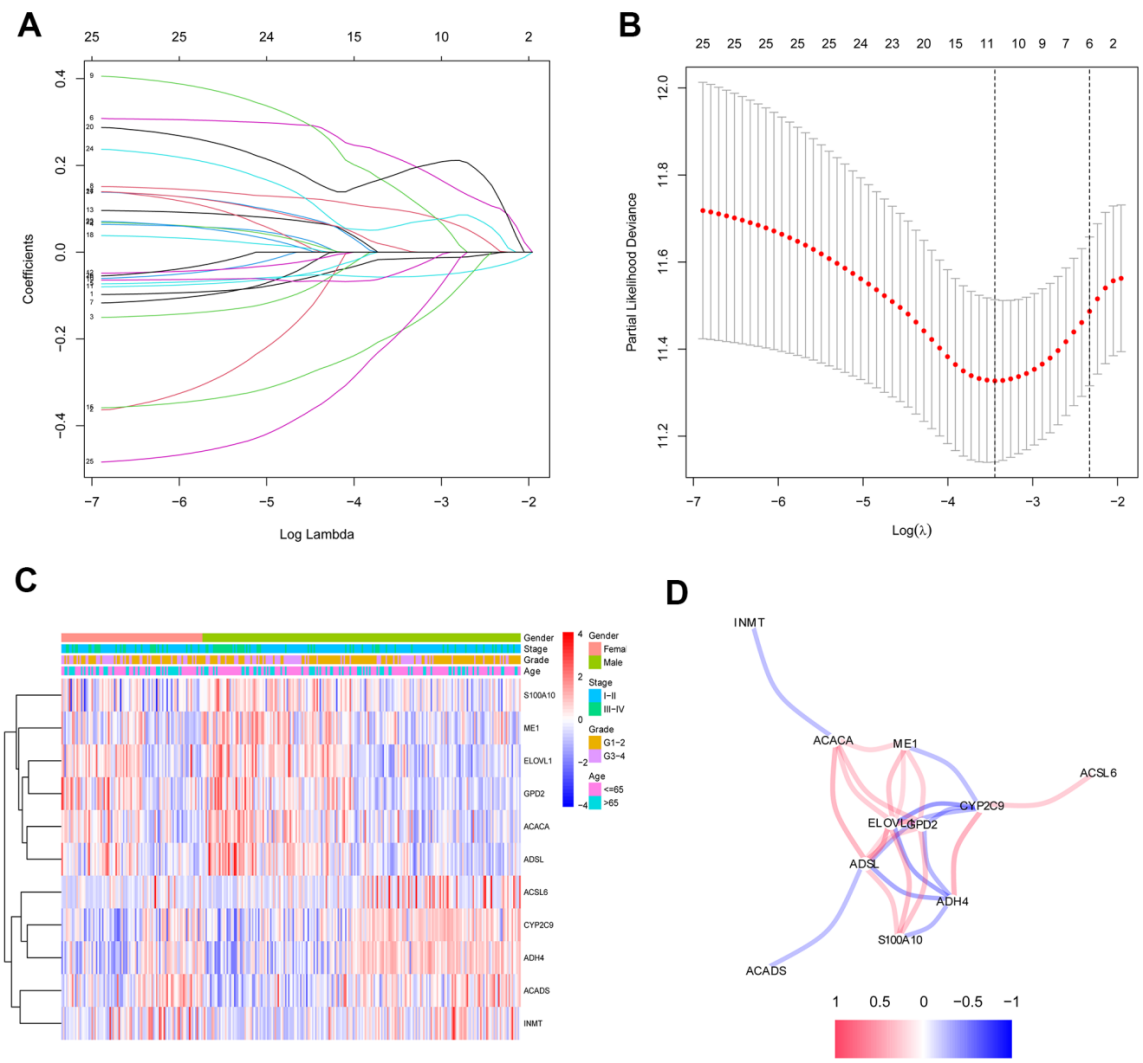
**Identification of fatty acid metabolic signatures by LASSO regression algorithm in HCC.** (**A**) LASSO coefficient profiles of the 26 fatty acid metabolic genes. (**B**) Cross-validation for tuning parameter selection in the proportional hazards model. (**C**) The heatmap of the 11 fatty acid metabolic genes. (**D**) The correlation of the 11 fatty acid metabolic genes.

**Figure 4:**
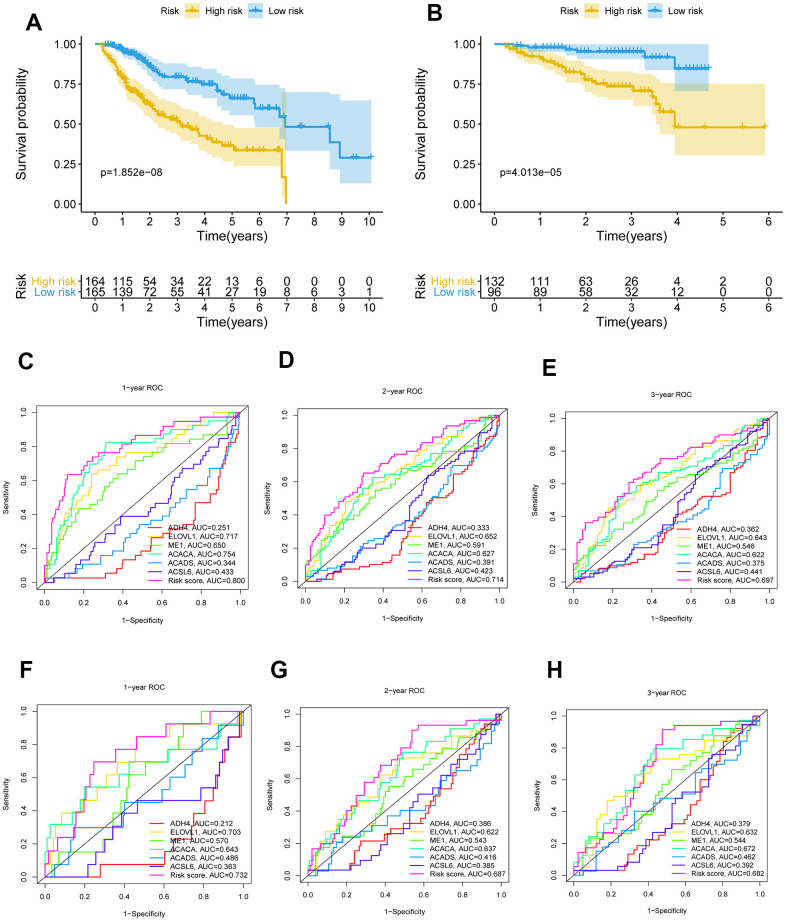
**K-M survival analysis and ROC curves of risk prognostic model in HCC patients.** (**A**, **B**) K-M survival analysis of risk prognostic model of HCC patients in TCGA and ICGC. (**C**–**E**) ROC curves analysis of risk prognostic model of HCC patients at 1 year, 2 years and 3 year in TCGA. (**F**–**H**) ROC curves analysis of risk prognostic model of HCC patients at 1 year, 2 years and 3 year in ICGC.

**Figure 5:**
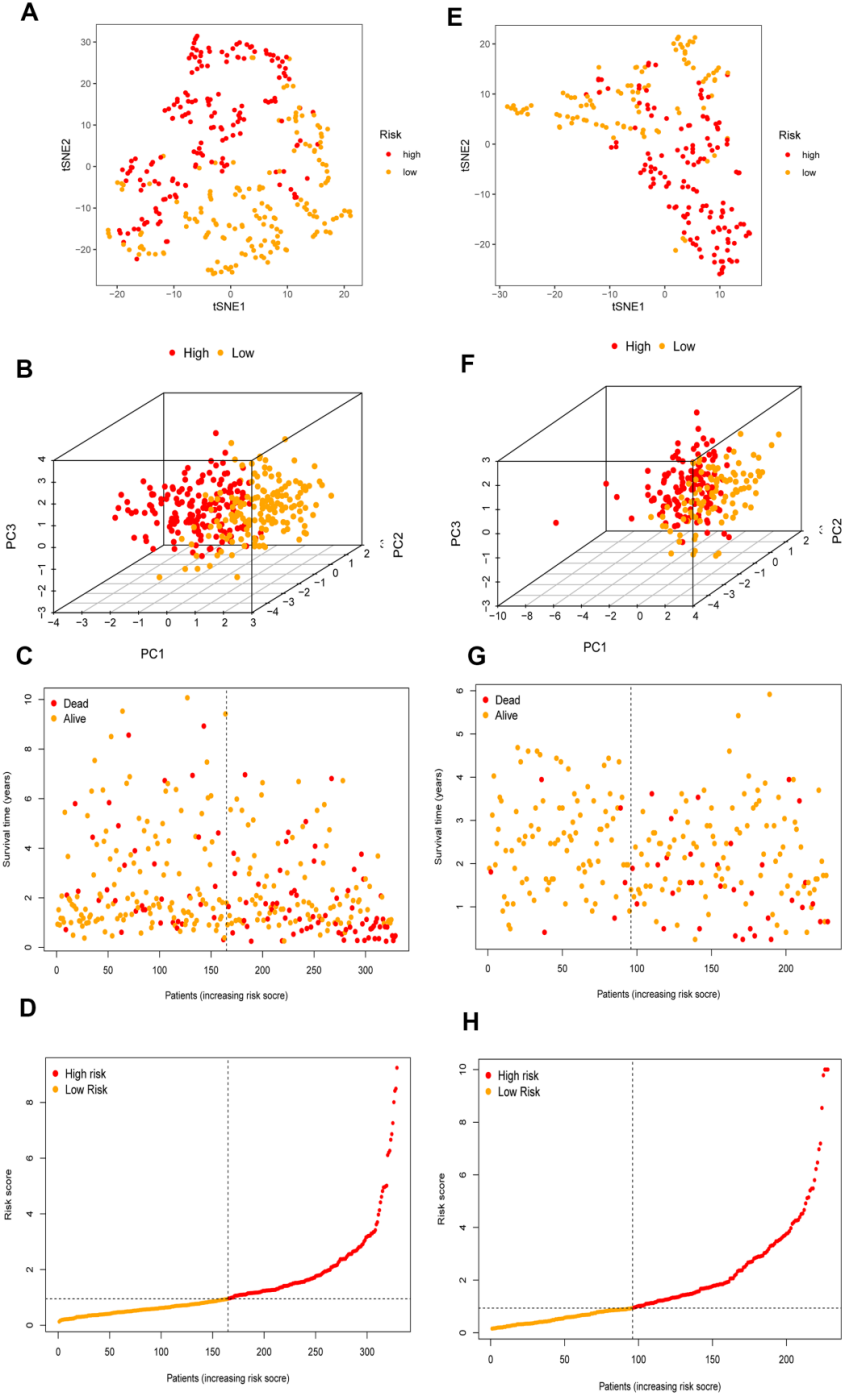
**The validation of the risk prognostic model in HCC patients.** (**A**–**D**) t-SNE, PCA, survival status scatter plots and risk score distribution shown the power prognostic ability of the risk prognostic model in TCGA. (**E**–**H**) t-SNE, PCA, survival status scatter plots and risk score distribution shown the power prognostic ability of the risk prognostic model in ICGC.

The 26 filtered genes were further included in the least absolute shrinkage and selection operator (LASSO) logistic regression algorithm (Figure 3A), cross-validation was conducted, and 11 prognostic signatures were screened to forecast an individual’s prognostic risk (Figure 3B). The heatmap of 11 genes is shown in Figure 3C, and the correlation network of the 11 genes is shown in Figure 3D. Then, 6 independent prognostic genes were further screened by performing a multivariable Cox regression analysis of the 11 prognostic signatures, and a prognostic model was constructed. Risk score = (-0.081×level of the *ADH4* mRNA)+(0.339×level of the *ELOVL1* mRNA)+(0.137× level of the *ME1* mRNA+(0.397×level of the *ACACA* mRNA)+(-0.330×level of the *ACADS* mRNA)+(-0.417×level of the *ACSL6* mRNA). Three hundred twenty-nine patients with HCC were separated into high-risk (n=164) and low-risk (n=165) groups by the median risk score as the cutoff value. The OS of the two groups was significantly different in the Kaplan-Meier (K-M) analysis (P<0.001) (Figure 4A). A receiver operating characteristic (ROC) curve was constructed to estimate the model and assess the reliability of the risk score, and the areas under the curve (AUCs) at 1 year, 2 years and 3 years were 0.800, 0.714 and 0.697, respectively (Figure 4C–4E). Moreover, principal component analysis (PCA) and t-distributed stochastic neighbor embedding (t-SNE) analysis were performed to identify low-risk patients and high-risk patients. As shown in Figure 5A–5D, patients with HCC were divided into different risk groups with a relatively clear resolution.

### Validation of the risk model in patients with HCC

To further validate the risk model, the risk model was used to assess patients in the ICGC database according to the median risk score of the 6 genes in TCGA. The P value for the K-M analysis of the high-risk and low-risk groups was less than 0.001 in the ICGC database (Figure 4B). Meanwhile, the AUCs for 1 year, 2 years and 3 years were 0.732, 0.687 and 0.682, respectively (Figure 4F–4H). PCA and t-SNE analysis were also conducted to test the risk model based on the ICGC database. As shown in Figure 5E–5H, high-risk and low-risk patients were divided by different analyses.

### Functional enrichment analysis of the screened genes

**Figure 6:**
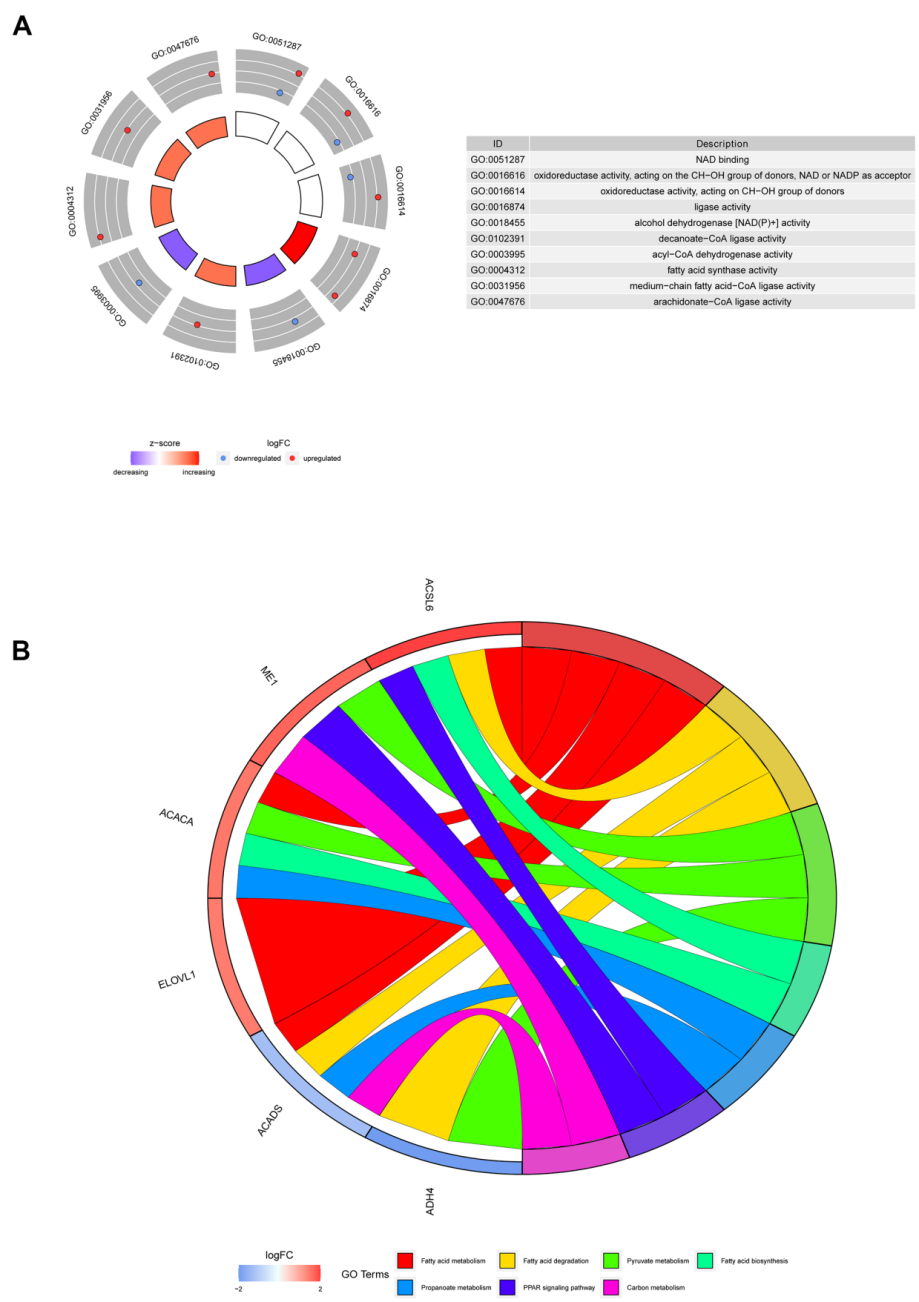
**Representative results of GO and KEGG analyses.** (**A**) The molecular functions of the 6 screened genes. (**B**) The potential biological pathways of the screened genes.

The most highly enriched molecular functions of the 6 screened genes were nicotinamide adenine dinucleotide (NAD) binding, oxidoreductase activity and fatty acid activity (Figure 6A). The involvement of these molecular functions in fatty acid metabolism has been previously reported [14, 15]. Moreover, the Kyoto Encyclopedia of Genes and Genomes (KEGG) pathway analysis suggested that the 6 genes might participate in pathways related to fatty acids (Figure 6B).

### Integrated analysis of the risk score and clinical parameters of patients with HCC

**Figure 7:**
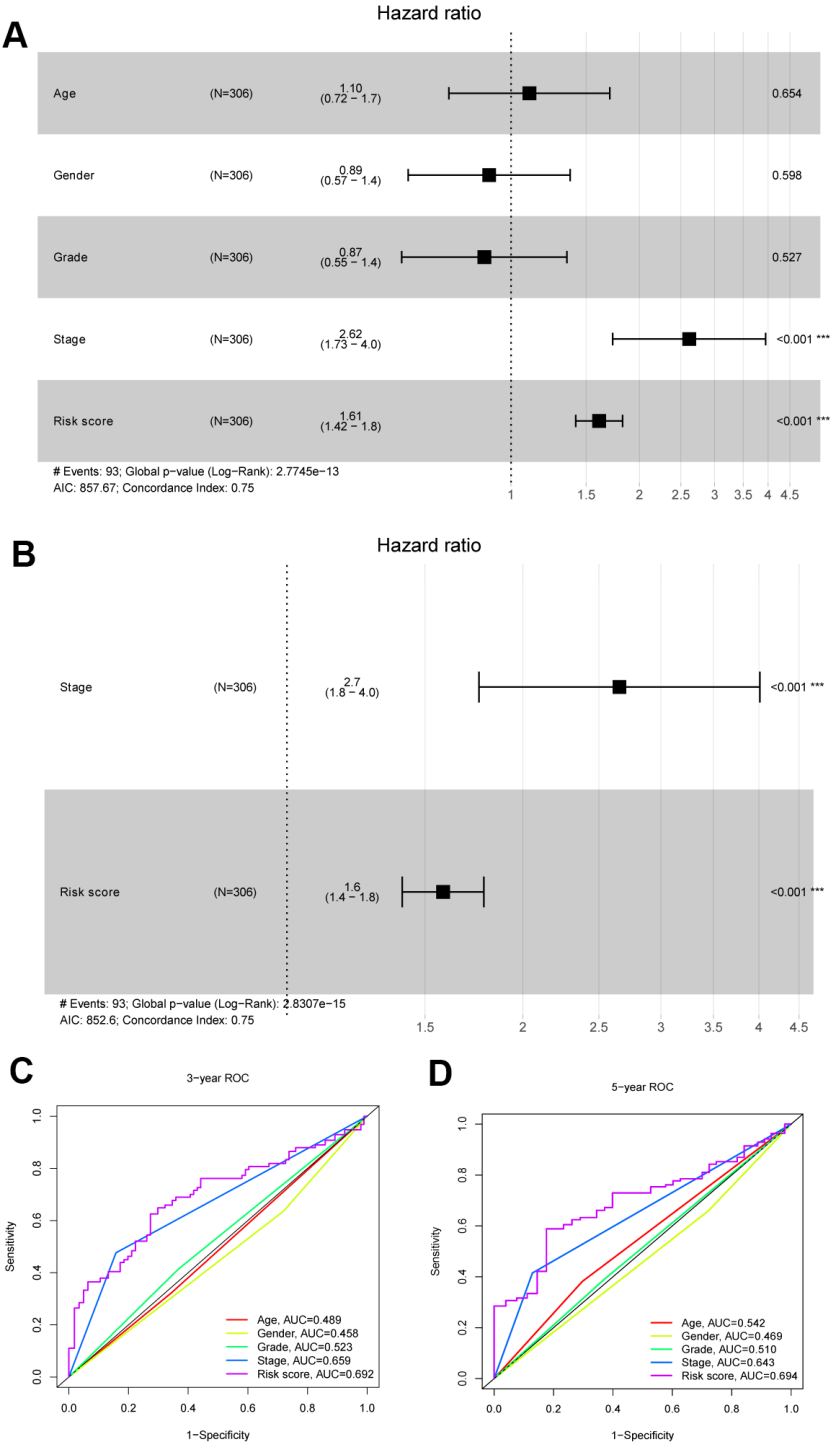
**Identification independent prognostic parameters in HCC patients.** (**A**, **B**) The univariate and multivariate Cox regression analysis of clinical parameters in patients with HCC. (**C**, **D**) ROC curves of risk score and clinical characteristics predicting 3- and 5-year survival in HCC patients.

Comprehensive data were extracted for 306 patients by integrating the clinical data and risk score of patients with HCC in TCGA database. First, univariate Cox regression analyses were conducted to filter the parameters (age, sex, stage, grade and risk score), and two factors (stage and risk score) were identified to be correlated with the OS of patients with HCC (P<0.001) (Figure 7A). Then, the stage and risk score were suggested to be independent prognostic parameters of patients with HCC by the multivariable Cox regression algorithm (P<0.001) (Figure 7B). Figure 7C, 7D shows the highly sensitive and specific predictive performance of the risk score determined using the ROC curve analysis.

### Construction and assessment of the nomogram for patients with HCC

**Figure 8:**
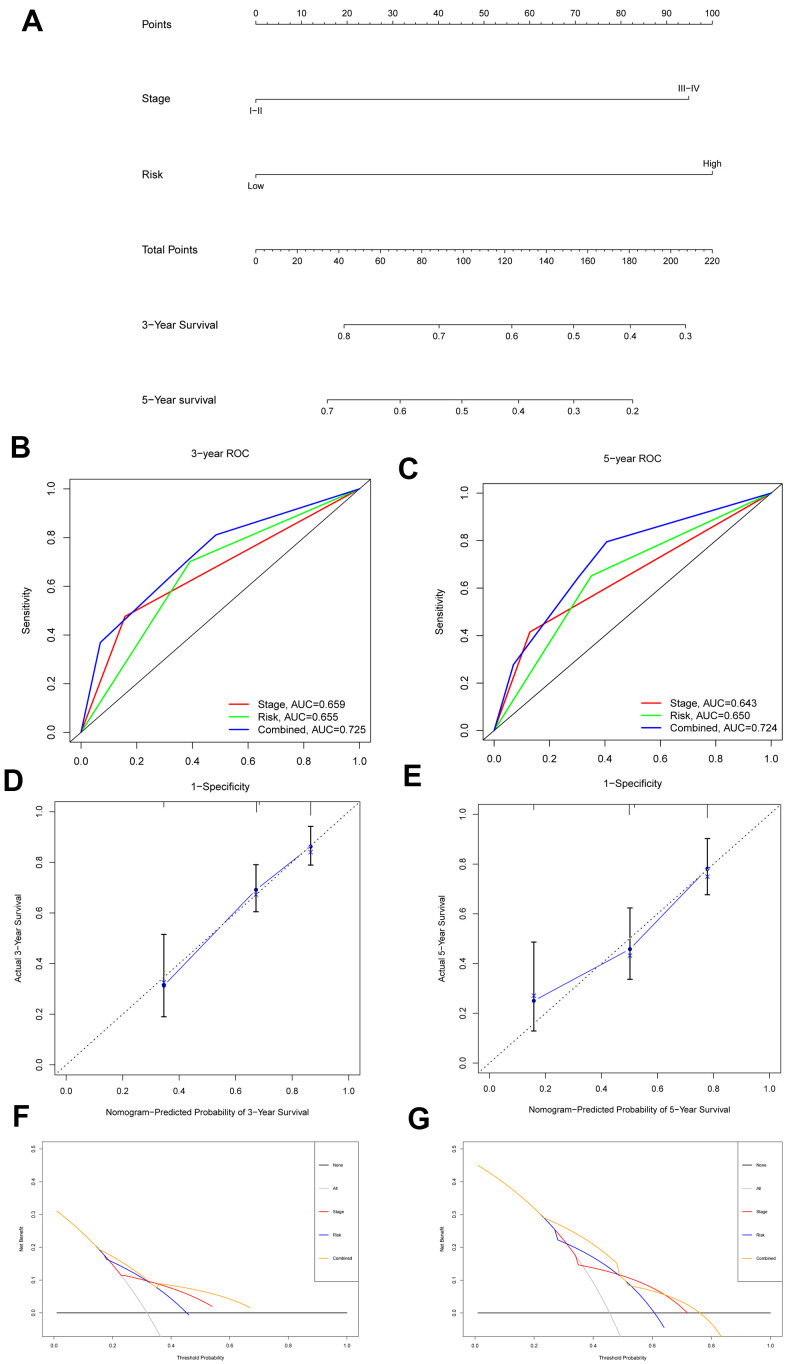
**Nomogram to predict 3- and 5- year OS and its validation in HCC patients.** (**A**) Nomogram to predict 3- and 5- year OS of HCC patients. (**B**, **C**) ROC curves to assess the accuracy of nomogram to predict 3- and 5- year OS in HCC patients. (**D**, **E**) Calibration plot analysis to assess the accuracy of nomogram to predict 3- and 5- year OS in HCC patients. (**F**, **G**) DCA to assess the accuracy of nomogram to predict 3- and 5- year OS in HCC patients.

The risk score and stage were included in the construction of a nomogram with an optimal concordance index (C-index, 0.709) to further analyze the ability of independent prognostic factors to estimate individual OS (Figure 8A). The application of the nomogram to patients in the ICGC database also showed a satisfactory predictive effect (C-index, 0.727). In addition, the performance of the nomogram in predicting the 3- and 5-year OS rates of patients with HCC was calculated. A ROC curve analysis was performed to validate the practicability of the nomogram, and the AUCs were computed for 3- and 5-year survival (0.725 and 0.724, respectively) (Figure 8B, 8C). The calibration plot was close to the ideal curve (Figure 8D, 8E). Moreover, decision curve analysis (DCA), a novel reliable evaluation tool to quantify clinical values, showed that the nomogram obtained a better net benefit than a single independent predictive parameter (Figure 8F, 8G). Collectively, the prognostic capacity of the tumor prognostic model was verified from multiple perspectives.

### Conjoint analysis of the tumor mutation burden and immune cell infiltration in patients with HCC

**Figure 9:**
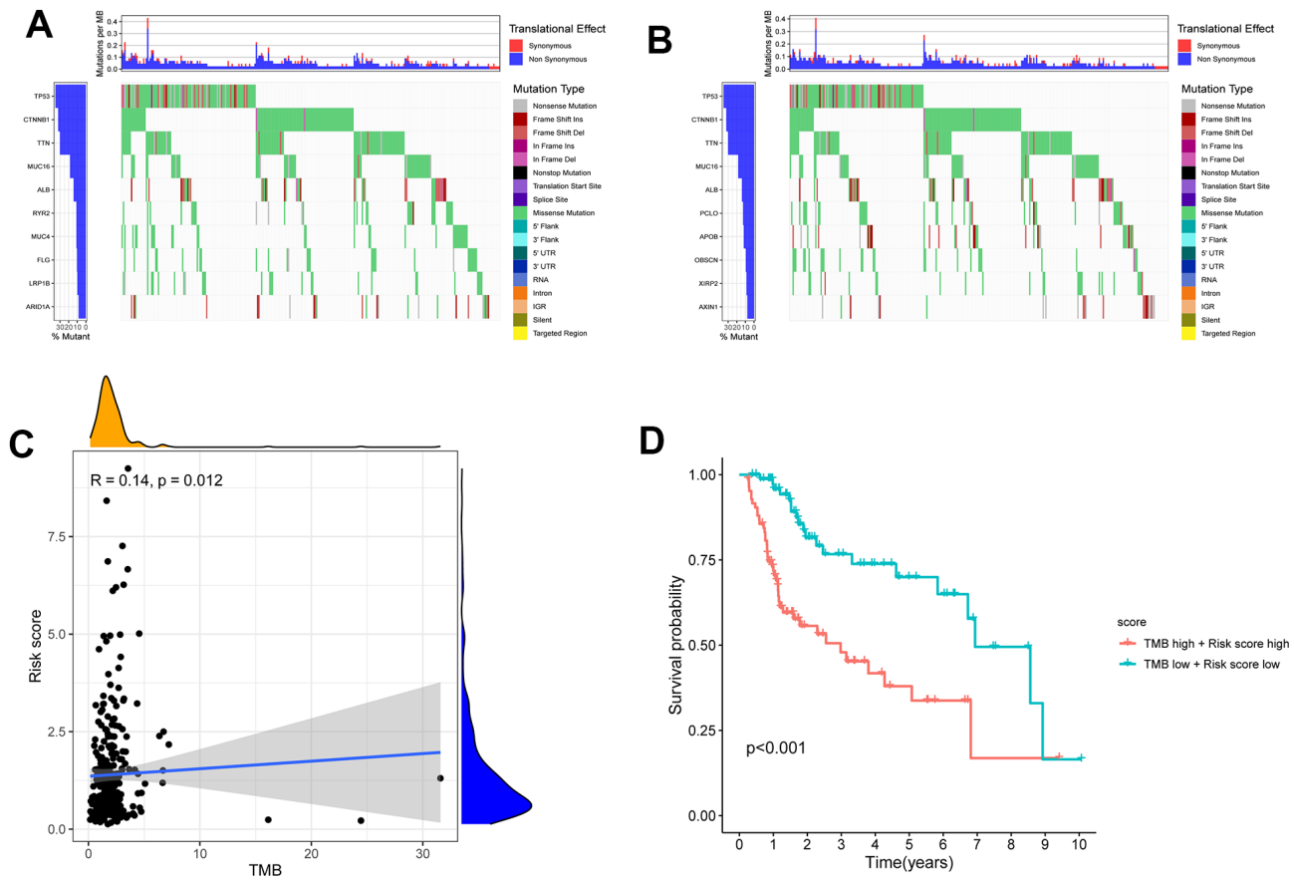
**The differences of TMB in high and low risk HCC patients.** (**A**) The TMB in high risk HCC patients. (**B**) The TMB in low risk HCC patients. (**C**) The TMB was positively correlated with the risk score in patients with HCC. (**D**) The OS of the HCC patients of high risk and high TMB were lower than those in HCC patients with low risk and low TMB.

The tumor mutation burden (TMB) has been suggested to be a marker for identifying patients with cancer who might benefit from immunotherapy and has been used to predict the curative effect of immune checkpoint inhibitors [16]. After intersecting the ID code of 364 HCC samples related to single nucleotide variation (SNV) and high- and low-risk patients with HCC, the SNV data of 156 high-risk HCC samples and 161 low-risk HCC samples were obtained. Figure 9A, 9B shows the difference in TMB in the high- and low-risk groups of patients with HCC. Moreover, the correlation analysis indicated that the TMB was positively correlated with the risk score (R=0.14; P=0.012) (Figure 9C). The 5-year survival rate of the low-risk and low-TMB group was better than that of the high-risk and high-TMB group (P <0.001) (Figure 9D).

**Figure 10:**
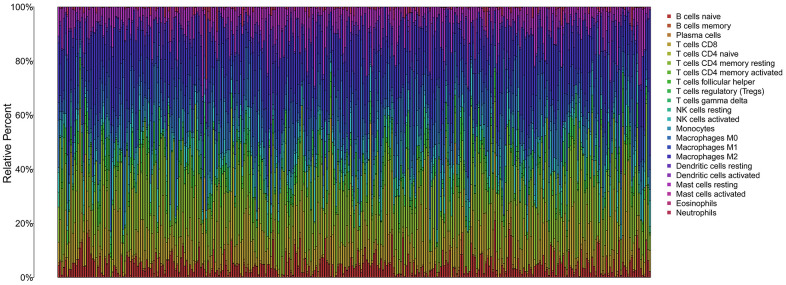
The immune infiltration of 22 immune cell types in high and low risk patients with HCC.

**Figure 11:**
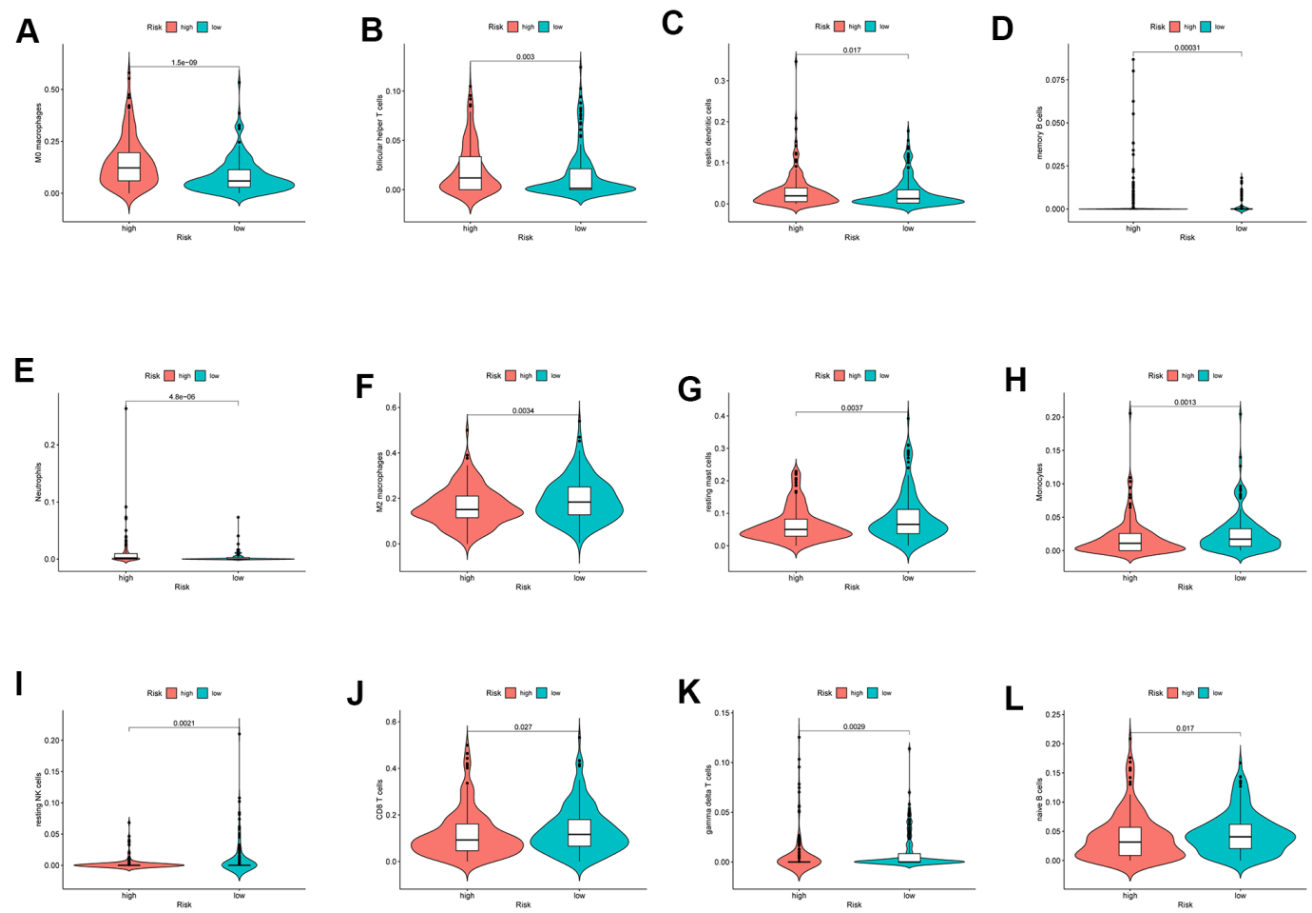
**The different immune infiltration in the high and low risk HCC patients.** (**A**–**E**) The expression of five types of immune cells is higher in high risk group compared with low risk group. (**F**–**L**) The expression of seven types of immune cells is higher in low risk group compared with high risk patients.

The infiltration of 22 immune cell types in high- and low-risk patients with HCC is shown in Figure 10, which suggested a potential difference in the immune microenvironment in patients with HCC. Moreover, different types of immune cells infiltrated different risk groups. In high-risk patients with HCC, higher expression of markers of M0 macrophages (P<0.001), follicular helper T cells (P=0.003), resting dendritic cells (P=0.017), memory B cells (P<0.001) and neutrophils (P<0.001) was observed (Figure 11A–11E), while lower expression of markers of M2 macrophages (P<0.01), resting mast cells (P<0.01), monocytes (P<0.01), resting NK cells (P<0.01), CD8 T cells (P=0.027), gamma delta T cells (P<0.01) and naive B cells (p=0.017) were observed than in the low-risk group (Figure 11F–11L).

## DISCUSSION

Convincing evidence has shown that one of the hallmarks of tumor cells, namely, metabolic dysregulation, contributes to abnormal cell biological behaviors, such as cell growth, angiogenesis, proliferation and invasion [4]. Abnormal glycolytic metabolism has already been confirmed in various tumors and has been widely used in clinical diagnosis and treatment [17]. Fatty acid metabolism, an important component of energy metabolism, has received increasing attention, and this metabolic pathway participates in energy production, membrane synthesis and signal transduction in tumor initiation and progression [7]. As a molecularly heterogeneous malignant tumor, the molecular characteristics of HCC are correlated with tumor biological behaviors [18]. Therefore, key molecular markers related to fatty acid metabolism in HCC must be identified.

Currently, gene signatures that correlate with the prediction of prognosis based on specific characteristics have become a hotspot in cancer research [19–21]. To the best of our knowledge, we have built a novel risk prediction model for HCC based on fatty acid metabolism-related genes and further constructed a clinical predictive model for patients with HCC that might be used to assess the individual’s prognosis.

In the present study, we first systematically investigated fatty acid metabolism-related genes in patients with HCC. The 26 fatty acid metabolism DEGs related to OS in patients with HCC were identified with strict filter standards, and the TCGA and ICGC databases were used for validation. Furthermore, the LASSO regression algorithm was used to further filter the core genes related to fatty acid metabolism in patients with HCC. Multivariable Cox regression analysis was further used to identify 6 independent prognostic signatures, and a risk score model was constructed. The LASSO regression algorithm and multivariable Cox regression analysis are beneficial to avoid overfitting and improve the clinical practicability of the prognostic model.

Most of the 6 signature genes have been suggested to be involved in multiple cancers. However, the roles of these genes in determining the prognosis of HCC remain to be explored. *ADH4* and *ACADS* were previously suggested to be related to HCC. Alcohol dehydrogenase 4 (*ADH4)* is mainly correlated with ethanol oxidation in the presence of intoxicating levels of alcohol and hydroxyl fatty acids [22, 23]. A few studies reported that *ADH4* might be a tumor suppressor and independent prognostic factor for HCC [24]. Our study again verified this speculation and further explored the ability of the gene to predict the risk score of patients with HCC. ACADS is a key short variant of the acyl-CoA dehydrogenase (ACAD) gene family and might be a methylation biomarker in HCC. The expression level of ACADS was negatively correlated with the DNA methylation level [25]. However, as a key metabolic enzyme, the role of ACADS in fatty acid metabolism in patients with HCC is unclear. The present study explored the role of ACADS in fatty acid metabolism in patients with HCC and included it in the construction of a risk score model.

In addition, elongation of very-long-chain fatty acids 1 (*ELOVL1*) is a single elongase catalyzing the synthesis of both saturated very-long-chain fatty acids (VLCFAs) and monounsaturated VLCFAs [26]. ELOVL enzymes, including *ELOVL1*, play different roles, possess characteristic substrate specificities and show differential expression patterns in mammalian tissues. *ELOVL1* is expressed in multiple organs, including the lungs, kidney, skin and stomach, and dysfunctional ELOVL1 proteins have been suggested to be correlated with different types of human diseases [27, 28]. *ELOVL1* has been suggested to be involved in various diseases, including cancers. However, the role of this gene related to fatty acid metabolism in HCC has not been reported in previous studies. In the present study, *ELOVL1* was upregulated. The gene correlated with OS and was included in the construction of the prognostic risk model for patients with HCC, which further suggested the importance of the roles of the gene in the origination and development of HCC. Thus, the genes related to fatty acid metabolism might be potential therapeutic and prognostic targets in HCC, and further exploration of the relationships between the genes and HCC is warranted.

After a risk predictive model was constructed based on the selected 6 genes, ROC curve, PCA and t-SNE analyses were together used to suggest that patients with HCC could be divided into high- and low-risk groups with an optimistic definition based on the model. Then, the risk score was integrated with the clinical parameters (age, sex, stage, and grade) of patients with HCC, and the risk and stage were identified as independent prognostic factors, which further indicated the practicability of the model.

Next, we included the risk score based on the core fatty acid metabolism-related genes and stage to construct a tumor predictive model, and a nomogram was produced. The accuracy of the nomogram in calculating an individual’s prognosis was assessed using the C-index, ROC curve, calibration plot analysis and DCA. DCA is a novel method for evaluating predictive models and better meets clinical requirements than a ROC curve analysis [29, 30]. The four validation methods together showed the practicability of the nomogram at different levels. The nomogram designed to predict the prognosis of patients with HCC was more accurate than the models reported in some previous studies with a better C-index (0.709 VS 0.676) and AUC for 3 years (0.725 VS 0.694) and 5 years (0.724 VS 0.667) [31, 32]. The construction of the model might help to identify patients with HCC and a poor prognosis and assist with the timely implementation of interventions.

Tumor immunotherapy is only effective for some patients with HCC, although the therapy has been shown to be a crucial therapeutic method by activating the body’s own immune system. In recent years, a high TMB has been suggested to be correlated with the curative effect of immune checkpoint inhibitor treatments [33]. Moreover, the level of fatty acids in the microenvironment affects the function and phenotype of infiltrating immune cells [12, 34]. Studies have suggested that the effect of immunotherapy might be improved by regulating fatty acid metabolism in patients with cancer. In the present study, 12 types of infiltrating immune cells were identified between high- and low-risk patients with HCC, which might be beneficial for individualized immunotherapy and further improvements in the therapeutic effect.

Certainly, the potential limitations of the study should be noted. First, the follow-up time of the validation dataset from the ICGC database was insufficient, and thus the nomogram based on TCGA database must be validated using additional external datasets. Second, the in-depth molecular mechanisms of the fatty acid metabolism-related genes, including the genes included in prognostic models, must be further verified in experimental studies. In addition, the study was based only on research data from public databases, which might contribute to selection bias. Therefore, a multicenter and large-scale study should be implemented to further validate the clinical utility of our model.

In summary, for the first time, this study established and validated a novel prognostic model based on 6 fatty acid metabolism-related genes and further constructed a clinical prognostic model for patients with HCC using strict standards. Moreover, the differences in infiltrating immune cells between high- and low-risk patients with HCC were explored, which might be helpful in providing a synergistic effect when administering treatments targeting fatty acid metabolism and immunotherapy. Therefore, our findings suggest that the novel fatty acid-related gene signature might be beneficial to the development of individualized treatments and improve the OS of patients with HCC.

## MATERIALS AND METHODS

### Patients and clinical data collection

Messenger RNA (mRNA) sequencing data from patients with HCC were obtained from TCGA database (https://portal.gdc.cancer.gov/) (374 HCC samples and 50 normal liver samples) and the ICGC database (https://icgc.org/) (243 HCC samples and 202 normal liver samples). In addition, the clinical information of patients with HCC (n=377, and 260, respectively) was downloaded from the TCGA and ICGC databases.

### Acquisition of fatty acid metabolism-related genes in patients with HCC

Three gene sets related to fatty acid metabolism (Hallmark fatty acid metabolism genes, KEGG fatty acid metabolism pathways, and Reactome fatty acid metabolism genes) were acquired from the Molecular Signature Database v7.2 (MSigDB), and fatty acid metabolism-related genes were retrieved after overlapping genes were removed [35]. The “Limma” R package was used to filter DEGs with a false discovery rate (FDR) <0.05 and fold change >2 [36].

### Construction and assessment of the risk score predictive model

The expression profile of fatty acid metabolism-related genes was extracted from the sequencing data of HCC and nontumor liver tissues in TCGA. After excluding patients with a follow-up time of less than 90 days, the survival data were integrated with sequencing data for fatty acid metabolism-related genes from patients with HCC in TCGA database. A univariate Cox regression analysis was used to screen fatty acid metabolism-related genes associated with OS in patients with HCC (P<0.05), and then DEGs related to fatty acid metabolism were filtered by matching the DEGs in patients with HCC from TCGA.

To further improve the accuracy, the filtered fatty acid metabolism-related genes in patients with HCC were proven to be DEGs in the ICGC database. To avoid overfitting, the LASSO logistic regression algorithm was used to further filter core fatty acid metabolism-related genes correlated with OS.

Moreover, multivariable Cox regression analyses were subsequently conducted to identify independent prognostic genes and construct a risk score predictive model for patients with HCC based on TCGA database. Then, the model was tested in the ICGC database to further validate the feasibility of the model. Moreover, multiple test methods were conducted. Patients with HCC were separated into high-risk and low-risk groups according to the median risk score, and the K-M method was used to analyze the difference in survival. ROC, PCA, and t-SNE analyses were used to further validate the feasibility of the risk score predictive model in patients with HCC.

### Functional enrichment analysis of the independent prognostic genes

Gene Ontology (GO) analysis is a major bioinformatics tool for annotating genes and gene functions [37]. KEGG is a collection of databases that contain information about genomes, biological pathways, diseases, and chemical substances [38]. The GO analysis and KEGG pathway enrichment analysis of the independent prognostic genes were conducted using the “clusterProfiler” package [39]. An FDR value<0.05 was considered statistically significant.

### Integrated analysis of the risk score predictive model and clinical parameters of patients with HCC

The following inclusion criteria for clinical factors were used to completely identify independent pretreatment predictors in patients with HCC: 1) neoadjuvant radiotherapy or chemotherapy was not received; 2) the follow-up time was more than 90 days; and 3) an operation was performed on all patients with HCC. The clinical information (age, sex, grade and stage) and risk score of patients with HCC (n=306) were integrated according to the ID of patients with HCC after excluding missing data. Then, univariate and multivariable Cox regression analyses were conducted to identify independent prognostic indicators among the clinical factors (P<0.05).

### Construction and assessment of the nomogram for patients with HCC

To further investigate individual OS, the “nomogram” R package was used to build a predictive model for patients with HCC based on the independent clinical parameters. The C-index, ROC curve, calibration plot and DCA were used to weigh the prognostic ability of the nomogram from multiple perspectives.

### Conjoint analysis of immune cell infiltration and the risk score in patients with HCC

The SNV data for patients with HCC were downloaded from TCGA database, and the TMB was calculated. Spearman’s correlation analysis was used to explore the correlation between the risk score and TMB. K-M analysis was used to analyze the 5-year survival rate between patients with a high TMB and risk score and patients with a low TMB and risk score. Moreover, the “Cell Type Identification by Estimating Relative Subsets of RNA Transcripts (CIBERSORT)” deconvolution algorithm with 1,000 permutations was applied to quantify 22 types of tumor-infiltrating lymphocytes (TILs) in the microenvironment of high- and low-risk patients with HCC [40].
